# Long-term sequelae and functional outcomes in the largest cohort of Nipah virus survivors in Bangladesh

**DOI:** 10.1016/j.lansea.2026.100729

**Published:** 2026-02-11

**Authors:** Wasik Rahman Aquib, Utpal K. Mondal, Arifa Nazneen, Dewan Imtiaz Rahman, Shadman Sakib Choudhury, Tonmoy Sarkar, Smita Sarker, S.M. Zafor Shafique, Ariful Islam, Arifur Rahman Bablu, Muhammad Rashedul Alam, Faruq Abdulla, Nazrul Islam, Md. Kamal Hossain, Ayesha Siddika, Carolyn Clark, Vijay Zala, Neil Cherian, Kamal Ibne Amin Chowdhury, Sharmin Sultana, John D. Klena, Mohammed Ziaur Rahman, Mustafizur Rahman, Stephen P. Luby, Joel M. Montgomery, Tahmina Shirin, Sayera Banu, Syed Moinuddin Satter

**Affiliations:** a Infectious Diseases Division, International Centre for Diarrhoeal Disease Research (icddr,b), Bangladesh, Dhaka, Bangladesh; b Rural Health Research Institute, Charles Sturt University, Orange, NSW, Australia; c University College London, London, United Kingdom; d Coalition for Epidemic Preparedness Innovations (CEPI), Oslo, Norway; e Institute of Epidemiology, Diseases Control and Research (IEDCR), Dhaka, Bangladesh; f Viral Special Pathogens Branch, Division of High Consequence Pathogens and Pathology, Centers for Disease Control and Prevention (CDC), Atlanta, GA, United States; g Division of Infectious Diseases and Geographic Medicine, Stanford University, CA, United States

**Keywords:** Nipah virus, NiV, Survivors, Sequelae, Bangladesh

## Abstract

**Background:**

Since 2001, Bangladesh has experienced recurrent outbreaks of Nipah virus (NiV) infection. While acute-phase manifestations are well documented, long-term clinical and functional sequelae poorly characterized.

**Methods:**

We conducted a cross-sectional assessment of 52 adult NiV survivors in Bangladesh between November 2021 and February 2022 to document the persistence, severity, and functional impact of post infection symptoms. Symptom history, clinical evaluation, and functional status were assessed using structured questionnaires and the Washington Group Extended Set on Functioning (WG-ES).

**Findings:**

All the survivors reported fever during acute illness, with frequent symptoms including headache (71%), severe weakness (67%), and altered mental status (44%). Following recovery, survivors reported a wide range of symptoms including sleep disturbance (58%), gait disturbance (54%), chronic fatigue (52%), memory and concentration difficulties (54%) and myoclonus (48%). Neurological, musculoskeletal, and respiratory symptoms persisted with varying severity and chronicity. Functional assessments revealed notable disability in several domains, particularly anxiety (48%), mobility (31%), and cognition (25%). Although post-infection symptoms were common, 65% of survivors met criteria for disability in at least one functional domain, and 48% had disabilities across multiple domains.

**Interpretation:**

These findings highlight the substantial and enduring burden experienced by NiV survivors in Bangladesh. The range and persistence of sequelae in this cohort appear broader than previously documented in Malaysia–Singapore survivor studies; however, methodological differences limit formal comparison. As the largest clinical and functional assessment of NiV survivors to date, this study provides essential evidence to inform long-term care strategies and underscores the need for survivor-focused rehabilitation and monitoring in outbreak-prone settings.

**Funding:**

This study was supported by the 10.13039/100016302Coalition for Epidemic Preparedness Innovations (CEPI) (Grant No: GR-02160). The funding body had no role in the study design; data collection, management, analysis, or interpretation; writing of the manuscript; or the decision to submit the manuscript for publication. The views and conclusions expressed in this manuscript are those of the authors and should not be construed as representing the official viewpoints of CEPI or the U.S. Centers for Disease Control and Prevention.

**Research in context**

**Evidence before this study**

We searched PubMed and Google Scholar using the terms “Nipah virus survivors,” “long-term outcomes,” “post-infection sequelae,” “disability,” and “neurological complications” with a focus on studies from Bangladesh, Malaysia, and Singapore, published up to April 2025. While a few studies have documented post-infection complications in small groups of Nipah survivors—particularly from Malaysia and Singapore—these were conducted within a narrow time frame post-infection and focused primarily on neuropsychiatric or imaging outcomes. In Bangladesh, limited studies have reported neurological sequelae, but no study has provided a comprehensive, multi-systemic assessment of clinical and functional outcomes across the full cohort of known survivors.

**Added value of this study**

This is the largest and most comprehensive study of Nipah virus survivors to date, assessing individuals infected across two decades (2001–2021) in Bangladesh—a country that experiences near-annual outbreaks. The study systematically evaluated new-onset physical, neurological, and functional symptoms, including disability, using standardized tools. It reveals a broader and more chronic pattern of post-infection sequelae compared to survivors in Malaysia and Singapore. This research provides updated insights into the survivor experience of NiV-B strain infections and underscores the need for structured follow-up and care programs.

**Implications of all the available evidence**

The findings suggest that long-term outcomes among survivors of Nipah virus infection may vary by strain, with NiV–B (circulating in Bangladesh) potentially associated with more severe and chronic post-infection sequelae. This highlights the importance of integrating survivor care into national outbreak preparedness plans. It also points to the need for strain-specific follow-up research, as well as development of tailored rehabilitation programs and mental health services in endemic regions.

## Introduction

Nipah virus (NiV) is an emergent zoonotic pathogen first identified during the late 1990s in the Malaysia–Singapore region. This fatal disease has caused outbreaks in five Southeast Asian nations since then, especially in Bangladesh and India.^1^, ^2^, ^3^, ^4^ NiV infection is marked by severe encephalitis, respiratory disease, and high mortality rates, exhibiting considerable variability in clinical presentations and significant human-to-human transmission.^5^, ^6^, ^7^, ^8^ Based on the evidence of human-to-human transmission, the potential to cause public health emergencies and the absence of efficient drugs or vaccines against NiV, the World Health Organization (WHO) declared NiV as one of the priority pathogens for research and development in 2018.^9^

NiV infection typically manifests as acute encephalitic syndrome characterized by fever, headache, dizziness, altered mental status, seizures, and rapid neurological deterioration, as observed during both the Malaysia–Singapore outbreaks and subsequent epidemics in Bangladesh.^3^^,^^8^^,^^10^, ^11^, ^12^ Respiratory involvement, ranging from cough and dyspnea to severe respiratory distress, has also been observed with varying frequency across outbreaks.^8^^,^^10^ In addition to this, NiV has also been documented to cause relapsing or late-onset encephalitis, occurring months to years after recovery, reflecting its potential for viral persistence or recrudescence.^13^^,^^14^ These acute and delayed clinical features underscore the need for greater understanding of the long-term health trajectory of survivors.

While NiV is widely recognized as a high-consequence pathogen due to its outbreak potential, the long-term morbidity among survivors represents an additional and underappreciated burden.

Studies from Malaysia and Singapore have documented persistent neurological deficits, cognitive impairments, motor dysfunction, psychiatric symptoms, and late-onset encephalitis among NiV survivors.^11^, ^12^, ^13^, ^14^, ^15^, ^16^ Smaller studies from Bangladesh have similarly reported chronic headaches, myoclonus, behavioral changes, neurocognitive challenges, fatigue, and other long-term health complaints.^2^^,^^8^^,^^10^ However, these studies included limited sample sizes and did not systematically assess functional capacity or disability, leaving significant gaps in understanding the full spectrum of survivors’ long-term health outcomes.

Previous investigations by Sejvar et al. (2007) and Ng et al. (2004) have significantly contributed to our understanding of post-infection outcomes of NiV, offering a preliminary insight into the neurological sequelae and functional limitations faced by survivors.^14^^,^^15^

In this context, the present study aims to provide an updated, comprehensive assessment of long-term clinical outcomes and functional disability among NiV survivors in Bangladesh. By evaluating the chronicity and severity of post-infection symptoms and applying a standardized functional assessment tool across the largest survivor cohort studied to date, this work seeks to address critical knowledge gaps and inform long-term care strategies for individuals affected by NiV infection.

## Methods

Nipah virus infection survivors in Bangladesh: Till 2021, a total of 322 Nipah virus cases had been detected in Bangladesh through the national surveillance system, including 241 laboratory-confirmed cases and 81 probable cases. Case definitions followed the standardized IEDCR–icddr,b national surveillance criteria. A confirmed Nipah virus case was defined as an individual with laboratory evidence of infection, either by detection of NiV RNA using qRT-PCR or by detection of anti-NiV IgM antibodies by ELISA. A probable case was defined as a patient with clinical illness compatibale with NiV infection, who had an epidemiological link to a confirmed case but for whom specimen collection and laboratory confirmation were not possible—most commonly due to death prior to sample collection. These definitions have been detailed previously by Satter et al.^3^

As of 2021, Bangladesh has 85 documented survivors of Nipah virus infection (as defined in Table 1). These individuals are continuously monitored through the National Nipah Virus Surveillance program of Bangladesh, a joint initiative by the Institute of Epidemiology, Disease Control and Research (IEDCR) and icddr,b that has been ongoing since 2006.

**Table 1:** Trait and criterion required in the definition of Nipah virus infection survivor.

Trait	Description
A. Symptomatic evaluation, contact history, or presence in a Nipah virus outbreak area	Known history of fever (>38.5 °C) with new onset of altered mental status or new onset of breathing difficulty corresponding to a Nipah virus outbreak or a confirmed NiV case detected by surveillance^17^
	Contact with a known patient infected by NiV
	Presence in an area known to be affected by NiV at time of a known outbreak
B. Laboratory Confirmation	NiV RNA positivity in any specimen by NiV RT-PCR
	NiV IgM or IgG antibody positivity in serum, by anti-NiV human IgM/IgG ELISA

Definition of Nipah virus infection survivor: An individual was considered a Nipah virus infection survivor only if the individual matched one criterion under trait “A” and one under trait “B” in Table 1 and, if the individual has survived the acute infection.

Study sites and population: This cross-sectional study was conducted from November 2021 to February 2022, covering 23 of Bangladesh's 64 districts. This study was nested within a larger Nipah survivor cohort investigation that contributed to the development of the First WHO International Standard for anti–Nipah virus antibody.^18^ For that overarching study objective, collection of relatively large volumes of venous blood from each participant was required to generate sufficient serum for immunological analyses and reference reagent development. In accordance with WHO guidelines for donor suitability and institutional ethical requirements, such volumes could be collected only from adults ≥18 years of age.^19^ From the identified 85 Nipah virus infection survivors, 54 adult survivors were initially screened for participation, while the remaining 31 individuals were excluded due to the minimum age requirement for participation. After excluding those with ongoing acute febrile illnesses, 52 were enrolled in the parent study as well as this nested clinical assessment study. Thus, eligible participants were documented Nipah virus infection survivors aged ≥18 years, clinically stable at the time of assessment, and able to provide informed written consent. These survivors, dispersed across various districts, were evaluated in groups either at their nearest sentinel hospitals, part of the national Nipah virus surveillance network, or at the icddr,b hospital in Dhaka (see Appendix).

### Data collection

A structured questionnaire was administered by trained physicians to document basic demographic information and the history of acute illness during the initial Nipah virus infection. Because acute-phase information was already recorded through the national Nipah surveillance platform, responses were cross-checked against surveillance records to ensure accuracy and consistency.

For post-infection outcomes, physicians conducted structured, face-to-face clinical history-taking to document the current health status of survivors, with specific attention to symptoms and conditions that developed after acute infection. This assessment used a standardized symptom checklist covering general health, respiratory and cardiovascular systems, and neurological and musculoskeletal systems. Physicians verified each reported symptom through follow-up probing and clinical judgement, and documented all findings in a structured case assessment form. The chronicity and severity of symptoms were also recorded (Table 2). To ensure consistency across assessors, all physicians used the same standardized instruments and received uniform training in symptom elicitation and documentation.

**Table 2:** Operational definition of severity and chronicity of illness.

Severity/chronocity	Operational definition
**Severity of illness**	
Mild	A health condition that is present but tolerable, not affecting activities of daily living
Moderate	A health condition that could not be ignored, affecting activities of daily living
Severe	Worst possible health condition that could be imagined
**Chronicity of illness**	
Acute	Health condition that develops rapidly and resolves within 1 month
Episodic	A health condition that is transient, not lasting for more than 6 months at a stretch with disease- free period in between two consecutive episodes
Chronic	A health condition lasting for 6 months or more, that requires ongoing medical attention, or limits activities of daily living, or both

Clinical and functional assessment of NiV survivors: Upon the documentation of new onset symptoms, a thorough clinical evaluation of each survivor was performed by a neurologist. In addition, functional disability was assessed using the Washington Group Extended Set of Questions (WG-ES), which evaluates ten domains of functioning.^20^ The WG-ES was administered by trained physicians following standard administration guidelines, and responses were documented item-by-item according to established scoring procedures. Narrative documentation, clinical examination findings, and WG-ES assessments were cross-checked for internal consistency.

### Statistical analysis

Two team members regularly checked, verified, and entered data into an Excel file. Quantitative data were analyzed using STATA (Version 15.0). For symmetric continuous data, mean and range were calculated; for asymmetric data, median and range were used. Frequency, proportion, and 95% confidence intervals were determined for categorical variables. The Clopper-Pearson exact method was used to calculate the 95% confidence intervals for proportions in categorical variables. Chi-square and Fisher's exact tests assessed differences in signs/symptomsby sex and administrative region (division). Due to the increasing trend of the signs/symptoms with the age, firth logistic regression modelling was performed to examine the association of sings/symptoms with the age in years at acute infection (≤21 years versus ≥22 years) and age in years during study period (≤25 years, 26–40 years, ≥41 years). Differences in the severity and chronicity of the signs/symptoms by sex, age at acute infection and during study period were determined using Chi-square and Fisher's exact tests. Disability status was evaluated for ten functional domains using the Washington Group Extended Set on Functioning (WG-ES). Prevalence for each domain, as well as single and multiple domains of disability, was calculated. Fractional polynomial logistic regression modelling was carried out to determine the association of lag-time from symptoms onset to hospital admission (exposure) and age at acute infection (confounder) with the single domain disability (response). The single domain disability was defined as wheather survivors had disability condition of at least one functional domain. Due to small sample size, 10% level of significance was considered as a cut-off of P-value.

This manuscript was prepared in accordance with the STROBE (Strengthening the Reporting of Observational Studies in Epidemiology) guidelines for cross-sectional studies.^21^ A completed STROBE checklist is provided in Supplementary Table S1.

### Ethics statement

Informed written consent was obtained from the study participants before enrollment, and only the participants who provided their written consent were enrolled in the study. Approval of the study protocol (Protocol No-18067) was obtained from icddr,b's Institutional Review Board (IRB). The icddr,b IRB has two separate committees: Research Review Committee (RRC) and Ethical Review Committee (ERC). Approval was secured from both committees before the commencement of study activities. The medical records and clinical information of acute infection were obtained from the National Nipah Virus Surveillance of Bangladesh which was being implemented under the study protocol no PR-2005-026.

### Role of funding source

This study was supported by the Coalition for Epidemic Preparedness Innovations (CEPI) (Grant No: GR-02160). The funding body had no role in the study design; data collection, management, analysis, or interpretation; writing of the manuscript; or the decision to submit the manuscript for publication. The views and conclusions expressed in this manuscript are those of the authors and should not be construed as representing the official viewpoints of CEPI or the U.S. Centers for Disease Control and Prevention.

## Results

### Demographic information and signs/symptoms during acute infection

52 survivors of Nipah virus infection took part in this study, more than half (54%) of whom were male. The participants’ median age was 35 years (range: 19–68 years) during the research whereas it was 23 years (range: 4–50 years) when they were infected. Fever was the most common (100% of survivors) symptom reported during their acute phase of infection, followed by headaches (71%) and severe weakness (67%). None of the survivors had pre-existing medical conditions.

### Sequelae of NiV infection

Headache and fever were the most common post-acute symptoms in our population, affecting 83% and 77% of the patients, respectively. Coughing was the most prevalent respiratory symptom (63%) and most people (54%) reported difficulty concentrating and remembering things, which may be a sign of more serious cognitive impairmentsMoreover, Table 3 indicates that a greater percentage of female respondents (71%) than male (39%) experienced persistent subjective memory/concentration problems. Based on the bivariate analysis, there was a significant difference in proportions between the male and female survivors for symptoms like exhaustion, leg weakness, persistent behavioral or personality changes, ongoing subjective memory/concentration issues, low mood, pain behind the eyes, difficulty sleeping, dyspnea, and weakness in the arms.

**Table 3:** Bivariate association of symptoms, signs and complaints during post infection phase with the survivor's sex, age during study period, age at acute infection, and administrative division.

Post infection signs, symptoms and complaints	Sex			Age in years during study period				Age in years at acute phase of illness			Division (Higher-level administrative region)					Total
	Female	Male	P-value	≤25 years	26–40 years	≥41 years	P-value	≤21 years	>21 years	P-value	Dhaka	Khulna	Rajshahi	Rangpur	P-value	
	n (% of total female survivors)	n (% of total male survivors)		n (% of total survivors aged ≤25 years)	n (% of total survivors aged 26–40 years)	n (% of total survivors aged ≥41 years)		n (% of total survivors aged ≤21 years)	n (% of total survivors aged >21 years)		n (% of total survivors lived in Dhaka)	n (% of total survivors lived in Khulna)	n (% of total survivors lived in Rajshahi)	n (% of total survivors lived in Rangpur)		n (% of all survivors)
	[95% CI]	[95% CI]		[95% CI]	[95% CI]	[95% CI]		[95% CI]	[95% CI]		[95% CI]	[95% CI]	[95% CI]	[95% CI]		[95% CI]
Fever	21 (88) [68, 97]	22 (79) [59, 92]	0.480	10 (83) [52, 98]	19 (79) [58, 93]	14 (88) [62, 98]	0.729	19 (79) [58, 93]	24 (86) [67, 96]	0.543	20 (91) [71, 99]	4 (57) [18, 90]	9 (69) [39, 91]	10 (100) [69, 1]	0.037	43 (83) [70, 92]
Headache	18 (75) [53, 90]	22 (79) [59, 92]	0.761	11 (92) [62, 100]	17 (71) [49, 87]	12 (75) [48, 93]	0.375	19 (79) [58, 93]	21 (75) [55, 89]	0.741	20 (91) [71, 99]	3 (43) [10, 82]	8 (62) [32, 86]	9 (90) [55, 1]	0.019	40 (77) [63, 87]
Altered Mental Status	12 (50) [29, 71]	10 (36) [19, 56]	0.299	5 (42) [15, 72]	10 (42) [22, 63]	7 (44) [20, 70]	0.910	9 (38) [19, 59]	13 (46) [28, 66]	0.527	10 (45) [24, 68]	3 (43) [10, 82]	3 (23) [5, 54]	6 (60) [26, 88]	0.352	22 (42) [29, 57]
Loss of Consciousness	6 (25) [10, 47]	6 (21) [8, 41]	0.761	1 (8) [0, 38]	7 (29) [13, 51]	4 (25) [7, 52]	0.375	5 (21) [7, 42]	7 (25) [11, 45]	0.741	4 (18) [5, 40]	2 (29) [4, 71]	2 (15) [2, 45]	4 (40) [12, 74]	0.504	12 (23) [13, 37]
Seizures	5 (21) [7, 42]	5 (18) [6, 37]	0.786	1 (8) [0, 38]	4 (17) [5, 37]	5 (31) [11, 59]	0.148	4 (17) [5, 37]	6 (21) [8, 41]	0.688	4 (18) [5, 40]	2 (29) [4, 71]	0 (0) [0, 25]	4 (40) [12, 74]	0.068	10 (19) [10, 33]
Sensory loss or Changes	4 (17) [5, 37]	3 (11) [2, 28]	0.690	2 (17) [2, 48]	4 (17) [5, 37]	1 (6) [0, 30]	0.411	2 (8) [1, 27]	5 (18) [6, 37]	0.362	3 (14) [3, 35]	1 (14) [0, 58]	2 (15) [2, 45]	1 (10) [0, 45]	1.000	7 (13) [6, 26]
Depressed Mood	12 (50) [29, 71]	7 (25) [11, 45]	0.062	4 (33) [10, 65]	8 (33) [16, 55]	7 (44) [20, 70]	0.559	7 (29) [13, 51]	12 (43) [24, 63]	0.322	10 (45) [24, 68]	3 (43) [10, 82]	2 (15) [2, 45]	4 (40) [12, 74]	0.323	19 (37) [24, 51]
Pain behind the eyes	10 (42) [22, 63]	5 (18) [6, 37]	0.059	2 (17) [2, 48]	5 (21) [7, 42]	8 (50) [25, 75]	0.057	4 (17) [5, 37]	11 (39) [22, 59]	0.089	8 (36) [17, 59]	2 (29) [4, 71]	2 (15) [2, 45]	3 (30) [7, 65]	0.622	15 (29) [17, 43]
Blurry Vision	13 (54) [33, 74]	12 (43) [24, 63]	0.416	3 (25) [5, 57]	12 (50) [29, 71]	10 (63) [35, 85]	0.066	7 (29) [13, 51]	18 (64) [44, 81]	0.015	10 (45) [24, 68]	4 (57) [18, 90]	5 (38) [14, 68]	6 (60) [26, 88]	0.747	25 (48) [34, 62]
Oculomotor abnormalities	4 (17) [5, 37]	8 (29) [13, 49]	0.346	5 (42) [15, 72]	6 (25) [10, 47]	1 (6) [0, 30]	0.040	8 (33) [16, 55]	4 (14) [4, 33]	0.121	6 (27) [11, 50]	0 (0) [0, 41]	1 (8) [0, 36]	5 (50) [19, 81]	0.044	12 (23) [13, 37]
Hearing loss	3 (13) [3, 32]	2 (7) [1, 24]	0.652	0 (0) [0, 26]	1 (4) [0, 21]	4 (25) [7, 52]	0.055	1 (4) [0, 21]	4 (14) [4, 33]	0.284	3 (14) [3, 35]	1 (14) [0, 58]	0 (0) [0, 25]	1 (10) [0, 45]	0.526	5 (10) [3, 21]
Fatigue	17 (71) [49, 87]	10 (36) [19, 56]	0.012	6 (50) [21, 79]	8 (33) [16, 55]	13 (81) [54, 96]	0.074	9 (38) [19, 59]	18 (64) [44, 81]	0.062	15 (68) [45, 86]	3 (43) [10, 82]	1 (8) [0, 36]	8 (80) [44, 97]	0.001	27 (52) [38, 66]
Trouble sleeping	12 (50) [29, 71]	7 (25) [11, 45]	0.062	3 (25) [5, 57]	10 (42) [22, 63]	6 (38) [15, 65]	0.559	7 (29) [13, 51]	12 (43) [24, 63]	0.322	8 (36) [17, 59]	2 (29) [4, 71]	4 (31) [9, 61]	5 (50) [19, 81]	0.798	19 (37) [24, 51]
Cough	16 (67) [45, 84]	17 (61) [41, 78]	0.657	8 (67) [35, 90]	13 (54) [33, 74]	12 (75) [48, 93]	0.573	15 (63) [41, 81]	18 (64) [44, 81]	0.892	17 (77) [55, 92]	3 (43) [10, 82]	5 (38) [14, 68]	8 (80) [44, 97]	0.054	33 (63) [49, 76]
Breathing difficulty	8 (33) [16, 55]	3 (11) [2, 28]	0.086	2 (17) [2, 48]	2 (8) [1, 27]	7 (44) [20, 70]	0.074	2 (8) [1, 27]	9 (32) [16, 52]	0.057	6 (27) [11, 50]	2 (29) [4, 71]	0 (0) [0, 25]	3 (30) [7, 65]	0.120	11 (21) [11, 35]
Arm Weakness	14 (58) [37, 78]	9 (32) [16, 52]	0.058	5 (42) [15, 72]	9 (38) [19, 59]	9 (56) [30, 80]	0.409	8 (33) [16, 55]	15 (54) [34, 72]	0.155	11 (50) [28, 72]	2 (29) [4, 71]	3 (23) [5, 54]	7 (70) [35, 93]	0.118	23 (44) [30, 59]
Leg Weakness	13 (54) [33, 74]	6 (21) [8, 41]	0.015	4 (33) [10, 65]	7 (29) [13, 51]	8 (50) [25, 75]	0.336	4 (17) [5, 37]	15 (54) [34, 72]	0.010	8 (36) [17, 59]	2 (29) [4, 71]	5 (38) [14, 68]	4 (40) [12, 74]	1.000	19 (37) [24, 51]
Muscle pain	12 (50) [29, 71]	9 (32) [16, 52]	0.191	6 (50) [21, 79]	7 (29) [13, 51]	8 (50) [25, 75]	0.887	9 (38) [19, 59]	12 (43) [24, 63]	0.704	11 (50) [28, 72]	3 (43) [10, 82]	2 (15) [2, 45]	5 (50) [19, 81]	0.190	21 (40) [27, 55]
Joint Pain	12 (50) [29, 71]	12 (43) [24, 63]	0.606	5 (42) [15, 72]	9 (38) [19, 59]	10 (63) [35, 85]	0.246	10 (42) [22, 63]	14 (50) [31, 69]	0.557	12 (55) [32, 76]	3 (43) [10, 82]	7 (54) [25, 81]	2 (20) [3, 56]	0.309	24 (46) [32, 61]
Diarrhea	8 (33) [16, 55]	14 (50) [31, 69]	0.225	6 (50) [21, 79]	7 (29) [13, 51]	9 (56) [30, 80]	0.627	11 (46) [26, 67]	11 (39) [22, 59]	0.638	15 (68) [45, 86]	1 (14) [0, 58]	1 (8) [0, 36]	5 (50) [19, 81]	0.001	22 (42) [29, 57]
Abdominal pain	10 (42) [22, 63]	12 (43) [24, 63]	0.931	4 (33) [10, 65]	7 (29) [13, 51]	11 (69) [41, 89]	0.053	8 (33) [16, 55]	14 (50) [31, 69]	0.238	12 (55) [32, 76]	4 (57) [18, 90]	1 (8) [0, 36]	5 (50) [19, 81]	0.023	22 (42) [29, 57]
Weight loss	8 (33) [16, 55]	5 (18) [6, 37]	0.199	2 (17) [2, 48]	5 (21) [7, 42]	6 (38) [15, 65]	0.213	5 (21) [7, 42]	8 (29) [13, 49]	0.542	7 (32) [14, 55]	0 (0) [0, 41]	2 (15) [2, 45]	4 (40) [12, 74]	0.211	13 (25) [14, 39]
Persistent ataxia/gait disturbance	8 (33) [16, 55]	5 (18) [6, 37]	0.199	2 (17) [2, 48]	6 (25) [10, 47]	5 (31) [11, 59]	0.404	3 (13) [3, 32]	10 (36) [19, 56]	0.072	3 (14) [3, 35]	3 (43) [10, 82]	4 (31) [9, 61]	3 (30) [7, 65]	0.355	13 (25) [14, 39]
Persistent personality or behavioral changes	14 (58) [37, 78]	8 (29) [13, 49]	0.030	5 (42) [15, 72]	7 (29) [13, 51]	10 (63) [35, 85]	0.221	8 (33) [16, 55]	14 (50) [31, 69]	0.238	12 (55) [32, 76]	2 (29) [4, 71]	1 (8) [0, 36]	7 (70) [35, 93]	0.007	22 (42) [29, 57]
Persistent subjective difficulties with memory/concentration	17 (71) [49, 87]	11 (39) [22, 59]	0.023	6 (50) [21, 79]	11 (46) [26, 67]	11 (69) [41, 89]	0.294	10 (42) [22, 63]	18 (64) [44, 81]	0.112	15 (68) [45, 86]	3 (43) [10, 82]	3 (23) [5, 54]	7 (70) [35, 93]	0.043	28 (54) [39, 68]
Myoclonus	10 (42) [22, 63]	15 (54) [34, 72]	0.392	7 (58) [28, 85]	12 (50) [29, 71]	6 (38) [15, 65]	0.282	12 (50) [29, 71]	13 (46) [28, 66]	0.800	14 (64) [41, 83]	3 (43) [10, 82]	5 (38) [14, 68]	3 (30) [7, 65]	0.273	25 (48) [34, 62]

The bivariate analysis of post-infection symptoms in various age groups showed notable variations for several symptoms like exhaustion, breathing difficulties, abdominal discomfort, hearing loss, pain behind the eyes, blurry vision, and oculomotor abnormalities. Furthermore, some post-infection symptoms such as fever, headache, seizures, oculomotor abnormalities, fatigue, cough, diarrhea, abdominal pain, persistent personality or behavioral changes, and persistent subjective difficulties with memory/concentration significantly varied across administrative divisions.

### Chronicity and severity of sequelae

**Fig. 1:**
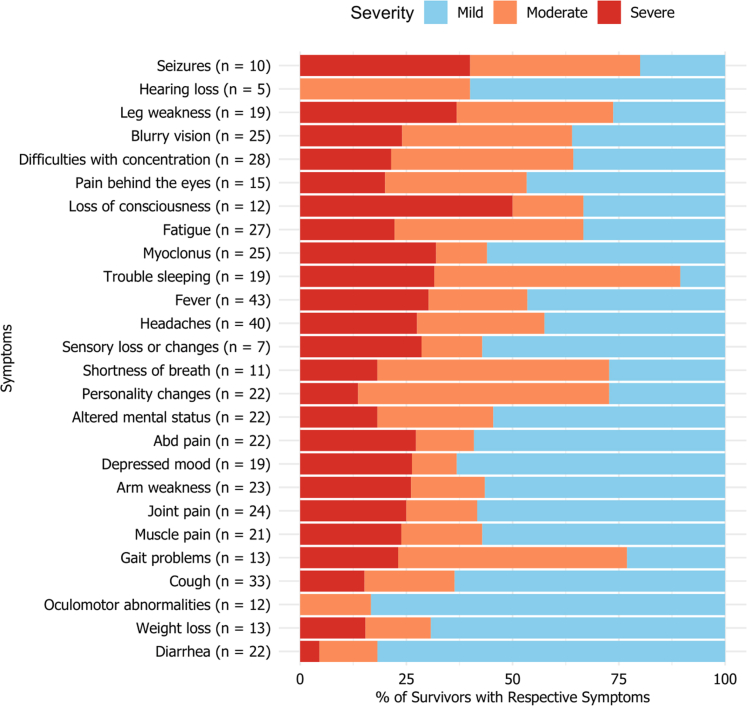
Sequelae among Nipah infection survivors in terms of severity of illness (“n” at the end of individual symptom/illness implies its prevalence among the 52 study participants).

**Fig. 2:**
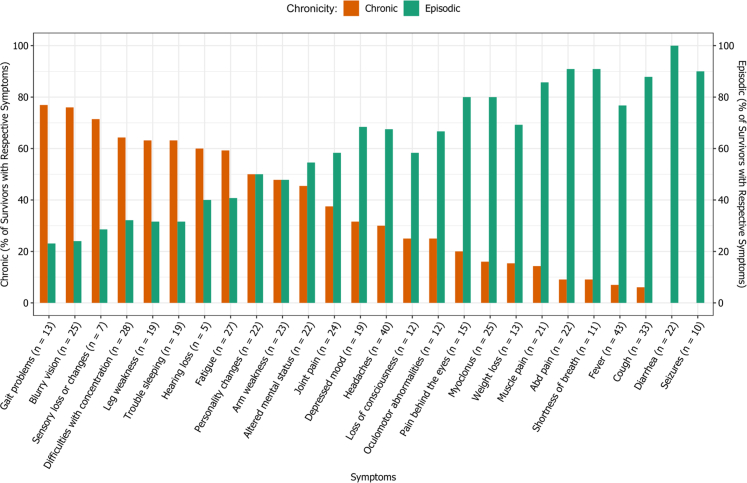
Sequelae among Nipah infection survivors in terms of chronicity of illness (“n” at the end of individual symptom/illness implies its prevalence among the 52 study participants).

The in-depth analysis of symptom severity among survivors post-Nipah virus infection, as depicted in Fig. 1, underscores a diverse and complex array of clinical manifestations. The most severe symptoms reported by the survivors were loss of consciousness, seizures, and leg weakness. In assessing the long-term impact of NiV infection, our study categorizes symptoms as acute, episodic, or chronic in alignment with the operational definitions outlined in the methodology (Supplementary Table S2). We found evidence of sustained neurological impairment with chronic manifestations such as gait problems, blurry vision, sensory loss or changes, concentration difficulty, and leg weakness, reported by over 60% of survivors (Fig. 2).

Conversely, Fig. 2 also visualizes the episodic symptoms with a high prevalence including depressed mood, headache, and pain behind the eyes, which may be reflective of the virus's impact on the vascular and affective components of the central nervous system.

Our study identified significant associations between sex and the severity of headache, fatigue, and memory/concentration difficulties, with these symptoms being more severe in female survivors. The analysis also showed significant associations between sex and the chronicity of altered mental status and personality or behavioral changes, with male survivors more frequently reporting chronic conditions.

### Assessment of functionality and disability

Although post-infection symptoms were common (Table 3), functional disability—measured using the WG-ES—was identified in a proportion of survivors (Table 4). Key observations include high levels of disability in terms of anxiety (48%), followed by mobility (31%) and cognition (25%).

**Table 4:** Functional disabilities among Nipah virus infection survivors (Based on WG-ES).

Functional domain	No difficulty	Some difficulty	A lot of difficulty	Cannot do at all	Disability
	n (%)	n (%)	n (%)	n (%)	n (%)
Cognition	21 (40)	18 (35)	3 (6)	10 (19)	13 (25)
Vision	23 (44)	21 (40)	7 (14)	1 (2)	8 (15)
Upper Body	29 (56)	11 (21)	11 (21)	1 (2)	12 (23)
Mobility	30 (58)	6 (12)	9 (17)	7 (13)	16 (31)
Communication	42 (81)	6 (12)	4 (8)	0 (0)	4 (8)
Hearing	47 (90)	2 (4)	3 (6)	0 (0)	3 (6)
Anxiety	4 (10)	17 (43)	7 (18)	12 (30)	19 (48)
Depression	5 (14)	24 (67)	4 (11)	3 (8)	7 (19)
Pain	25 (48)	15 (29)	9 (17)	3 (6)	12 (23)
Fatigue	33 (63)	15 (29)	3 (6)	1 (2)	3 (8)

Among 44 fractional polynomial logistic regression models, age was best represented by first- and second-order transformations (powers 1 and 2). The first-order age term was not significantly associated with single domain disability, however, the second-order age term had a significant non-linear relationship. Furthermore, although no statistically significant association was found between single domain disability and the time elapsed from symptoms onset to hospitalization, we observed that, a day's delay in hospitalization would increase the odds of reporting single domain disability by 18% (Table 5).

**Table 5:** Association of age at acute infection and the lag-time from symptoms onset to hospital admission with the single domain disability among survivors.

Acute-phase characteristics	Odds ratio (OR)	P-value	95% CI
Age in years (FP1: Age/10–2.3067)	0.06	0.157	0.001–2.92
Age in years (FP2: (Age/10)^2^–5.3207)	2.54	0.076	0.91–7.10
Lag time in days from symptoms onset to hospital admission (centered by 7)	1.18	0.218	0.91–1.53
Intercept	1.70	0.372	0.53–5.44

## Discussion

This study provides evidence of the sequelae of NiV infection among survivors, highlighting the spectrum of signs and symptoms that persist long after the initial recovery. The acute phase is characterized by a combination of fever, neurological symptoms, respiratory difficulties, and gastrointestinal disturbances. As survivors transition into the post-acute and long-term phase, the persistence of neurological, musculoskeletal, and psychological symptoms becomes evident. This points to the profound impact of the infection on overall health and well-being of survivors. Notably, the sequelae encompass a broad range of functional impairments including cognitive disabilities and mobility impairment, which substantially affect survivors’ quality of life. The diverse range of symptoms reported in this cohort also reinforces the need to consider Nipah infection as a condition with prolonged and multisystemic consequences rather than one confined to the acute phase.

Establishing a causal relationship between an acute infection and the development of sequelae through a cross sectional study is not possible, but our findings are consistent with previous reports from both Bangladesh and Malaysia.^14^, ^15^, ^16^^,^^22^ While it is impossible to exclude the possibility of an additional or concurrent infection, such as another form of viral encephalitis, none of the survivors reported fever or other signs of infection when the new neurological symptoms appeared. This makes it unlikely that another infection was responsible for these deficits. This alignment with earlier survivor studies strengthens the interpretation that the long-term manifestations observed here represent the enduring effects of the acute Nipah virus infection.

In our study, we observed a blend of neurological and neuropsychiatric sequelae in NiV survivors, reflecting the complex aftermath of the infection. Our findings generally align with previous studies from Bangladesh, but we noted a higher percentage of survivors suffering from focal weaknesses (arm and leg weakness), ataxia, personality/behavior changes, and memory difficulties compared to those reported by Sejvar et al.^14^ While Sejvar et al. found that 60% of survivors had fully recovered within two years of acute infection, our study found that 60% of survivors had at least one form of disability, irrespective of the years elapsed since acute infection.^14^ This notable difference underscores the need for further research to understand the factors contributing to the persistence of these disabilities, with a particular emphasis on exploring the potential changes in the epidemiology of sequelae. Additionally, unlike prior studies that primarily documented clinical symptoms, our assessment incorporated a standardized functional disability tool, allowing a more structured evaluation of the day-to-day impact of these symptoms. Our assessment of functionality and disability also sheds light on the severity and chronicity of post-infection symptoms, emphasizing the long-term impact on survivors' quality of life and highlighting the importance of improving existing rehabilitation programs to provide required care to the NiV survivors. Additionally, the high social burden imposed on caregivers, both primary and secondary, is often an unseen cost that should be considered when improving rehabilitation programs. Incorporating these costs and the needs of caregivers into the program can help ensure more comprehensive support for both survivors and their families.

A Malaysian study, conducted 10 years after their only recognized outbreak, included NiV encephalitis patients, non-encephalitis patients, and control groups, offering a broad comparison. Unlike Malaysia, which has experienced a single outbreak and has limited opportunities to follow survivors over time, Bangladesh experiences almost yearly outbreaks, allowing us to enroll survivors both recently infected and those infected decades ago.^3^ This provides a more comprehensive picture of post-infection sequelae and potential changes in long-term brain involvement. In contrast, our cohort predominantly consists of survivors with encephalitis, limiting intra-group comparisons but providing a thorough depiction of Bangladeshi survivors. Our study identified a broader range and higher prevalence of post-infection symptoms which is at least partly attributed to the fact that all of our participants had encephalitis; compared to only one third the participants of the study conducted by Sejvar et al.^14^ While the reporting of fatigue and focal neurological deficits was similar between the studies, other sequelae such as headache, memory impairment, loss/reduced consciousness reported in Malaysia, were less prevalent in our cohort. Additionally, we found several respiratory system-associated sequelae not reported in the Malaysian study, and a significantly higher percentage of survivors with disabilities.^16^ Differences in health-seeking behavior, access to acute-phase care, and the prolonged interval since infection in some Bangladeshi survivors may have also contributed to these variations.

The study conducted in Singapore, involving individuals infected during the Malaysia–Singapore outbreak, enrolled nine participants who underwent psychiatric evaluation, neuropsychological evaluation, and imaging.^16^ Conducted within two years of acute infection, this study provided an early perspective on post-infection sequelae. Unlike our study, which focused on a comprehensive evaluation of all existing survivors, the Singapore study robustly correlated imaging findings with neuro-psychological and psychiatric evaluations. Their study found a higher percentage of survivors suffering from clinical depression, with 3 of the 8 (38%) participants experiencing functional deficits or disabilities.^15^ This aligns with findings from Malaysia but is significantly lower than the prevalence observed in our study.^15^^,^^16^

Conversely, the studies from Malaysia and Singapore described notable neuropsychiatric outcomes, including mood disorders and cognitive impairments, underscoring the mental health implications of the infection.^15^^,^^16^ Our findings bridge these observations, revealing both similarities and differences in the sequelae spectrum. Unlike the focused neurological sequelae observed in Bangladesh, and the neuropsychiatric emphasis seen in Malaysia and Singapore, our study explored a broader range post-infection outcomes, encompassing physical and mental health impairments as well as functionality and disability related difficulties. This broader scope highlights the heterogeneity of long-term manifestations across regions and underscores the value of harmonized survivor follow-up frameworks.

Although Nipah virus outbreaks have occurred repeatedly in India, there is little to no publicly available information on the long-term clinical sequelae among Indian survivors. Detailed reports on acute infection, outbreak response, and mitigation efforts, as well as short- and long-term immunological outcomes, exist, but there is a lack of data on the clinical aftermath of infection in survivors.^23^^,^^24^ This gap highlights the need for further collaboration and research to better understand the long-term health impacts of Nipah virus infection in the Indian population. Follow-up studies of Nipah virus survivors from India would contribute valuable insights toward a more comprehensive understanding of the infection's long-term impact across different regional contexts.

Given that the Nipah virus has historically caused more fatal infections in Bangladesh and India compared to Malaysia–Singapore, and that the Malaysia–Singapore outbreaks were attributed to the NiV-M strain while almost all outbreaks in Bangladesh are linked to the NiV–B strain, our study raises important questions about potential strain-specific differences in post-infection sequelae.^3^^,^^25^, ^26^, ^27^ In terms of acute infection, prior studies have identified notable differences in pathogenicity and fatality rates between these strains in both animal and human models.^1^^,^^25^^,^^28^ In light of these findings, it is reasonable to hypothesize that strain variations could also influence the severity, persistence, and range of long-term sequelae. In addition, other reasons for these variations may include selection bias in follow-up, variations in the level of supportive care, and differences in the duration since infection. While our study does not directly establish a link between these factors and post-infection outcomes, the observed differences in the range and severity of sequelae between regions suggest that further investigation is warranted. Prospective, longitudinal studies with harmonized clinical, functional, and imaging assessments across countries would be essential to clarify these strain-specific or context-specific differences.

This research could provide deeper insights into the pathogenesis of Nipah virus and inform strategies for the long-term care of survivors. Despite Bangladesh being endemic for NiV and the critical importance of these unique individuals for future research on NiV-human interactions, there are currently no targeted programs or initiatives focused on ensuring their long-term comprehensive care. Additionally, with no licensed treatments or vaccines for Nipah virus to date, there remains a critical need for the development of effective medical countermeasures. CEPI's previous landscape assessment underscores the importance of advancing therapeutics, monoclonal antibodies, and vaccines to control future outbreaks.^29^ These efforts must be complemented by global collaboration and the establishment of targeted care and rehabilitation programs to address the specific needs of survivors and improve their quality of life.

Our study faced several limitations. A key limitation was the potential for recall bias in survivors’ recollections of acute-phase symptoms. Attempting to classify the Nipah virus infection as mild, moderate, or severe could have led to high chances of misclassification. Therefore, we chose not to classify the survivors based on their recollections of the acute infection. However, we mitigated this by matching our data with medical records and the database of the National Nipah Virus Surveillance of Bangladesh, which documented patients during their acute infection.

We focused on documentation of new symptoms following the acute infection, and most patients provided consistent responses during multiple lines of questioning, which supports the accuracy of our estimates. As this was a cross-sectional study, we recruited survivors at various time points post-infection (some infected 18 years ago and some 2 years ago). Because survivors were infected at different time points over nearly two decades, the study could not evaluate temporal changes or progression of sequelae. Our analyses therefore describe the presence and chronicity of long-term outcomes rather than their evolution over time.

Additionally, our study did not include imaging (MRI or equivalent) to observe the persistence of brain changes since infection, preventing us from correlating our findings with imaging results. The lack of standardized assessment during acute illnesses, particularly for survivors infected decades ago, made it difficult to categorize the severity of acute infection and correlate it with the extent of sequelae that developed. Proper documentation of the treatment each individual received would also have helped in correlating with the extent of sequelae they developed later on. Furthermore, evaluating current antibody titers (IgG and/or IgM) and comparing them with the clinical and functional findings could have added valuable insights.

Another key limitation of this study is the absence of a comparator group, which restricts our ability to determine whether the observed sequelae were specifically attributable to Nipah virus infection or reflect general post-infectious, post-hospitalisation, or age-related conditions. Additionally, comorbidity status was not systematically documented at the time of acute illness, limiting our capacity to distinguish new-onset sequelae from pre-existing conditions. These limitations should be considered when designing future studies and research on these unique but important individuals.

This study demonstrates the substantial long-term health consequences experienced by Nipah virus survivors in Bangladesh, with persistent neurological, physical, and functional impairments many years after infection. By documenting both symptom chronicity and disability using a standardized functional tool, this work provides the most comprehensive assessment of NiV survivors to date. The findings underscore the need for structured survivor follow-up, access to neurological and psychological support, and integration of rehabilitation services into routine care. While differences between regions and viral strains remain an important area for future investigation, longitudinal, harmonized studies will be essential to clarify how clinical and contextual factors influence long-term outcomes. Survivors of Nipah virus infection represent a uniquely vulnerable group, and ensuring their sustained clinical and functional care should be a public health priority.

## Contributors

Conceptualisation: SMS, WRA, UKM, DIR, MZR, NC.

Data curation: WRA, UKM, MRA, FA.

Formal analysis: WRA, FA, DIR, SMS.

Funding acquisition: SMS.

Investigation: SMS, WRA, UKM, SMZS, AA, ARB, MRA, NA, MKH, AS.

Methodology: SMS, SPL, UKM, WRA.

Project administration: SMS, SB, TS, SS, KIA, UKM, WRA, MZR.

Software: MRA, FA.

Supervision: SMS, UKM, WRA.

Validation: SMS, MZR, MR,

Visualisation: MRA, FA, WRA, DIR.

Data interpretation: WRA, UKM, DIR, NC.

Writing—original draft: WRA, UKM, DIR, MRA, FA.

Writing—review & editing: All authors.

SMS, UKM, WRA and DIR and have directly accessed and verified the underlying data reported in the manuscript.

## Data sharing statement

The data supporting the findings of this study are derived from an ongoing surveillance platform that has been operational for over 15 years and includes sensitive personal identifiers such as patient names, admission dates, locations, and GPS coordinates. At the time of consent, participants were assured that their identifiable information would remain confidential and would not be made publicly available. In accordance with icddr,b data protection policies and ethical obligations, the full dataset cannot be shared in the manuscript, supplemental files, or through a public repository, as it would risk disclosure of confidential information and unpublished data from ongoing studies.

However, de-identified data specific to this manuscript may be made available to qualified researchers upon reasonable request. Access will require approval from the icddr,b Data Repository Committee through a formal Data Licensing Application & Agreement. Additionally, as the surveillance platform is jointly operated with the Institute of Epidemiology, Disease Control and Research (IEDCR), Bangladesh, the Viral Special Pathogens Branch of the U.S. Centers for Disease Control and Prevention (US CDC), and CEPI, data sharing is also subject to clearance from all of the involved entities. Interested researchers should contact the Senior Manager, Research Administration & Strategy, icddr,b (shiblee_s@icddrb.org), or visit http://www.icddrb.org/for more information.

## Editor note

The Lancet Group takes a neutral position with respect to territorial claims in published maps and institutional affiliations.

## Declaration of generative AI and AI-assisted technologies in the manuscript preparation process

During the preparation of this work the author(s) used ChatGPT (OpenAI) in order to support language refinement, organization of reviewer responses, and improvement of clarity during manuscript revision. After using this tool/service, the author(s) reviewed and edited the content as needed and take(s) full responsibility for the content of the published article.

## Declaration of interests

No potential conflict of interest was reported by the author(s).
