# Supplementary material for: Long-term sequelae and functional outcomes in the largest cohort of Nipah virus survivors in Bangladesh
Source: Lancet Reg Health Southeast Asia. 2026 Feb 11;45:100729. doi: 10.1016/j.lansea.2026.100729 (PMC12915286; doi:10.1016/j.lansea.2026.100729)
Supplement: Supplementary Tables [file mmc1.docx]

Supplementary table 1: STROBE (Strengthening the Reporting of Observational Studies in Epidemiology) Checklist Cross-Referenced to Sections of This Manuscript

| **Item No.** | | **STROBE Recommendation** | **Where Addressed in Manuscript** |
| --- | --- | --- | --- |
| **Title and abstract** | 1(a) | Indicate study design in title/abstract | Abstract, Paragraph 2 (“…we conducted this cross-sectional study…”) |
|  | 1(b) | Informative, balanced summary | Entire Abstract—revised version reflects methods, key findings, and interpretation |
| **Introduction** | | | |
| Background  /rationale | 2 | Explain scientific background and rationale | Introduction, Paragraphs 1–4 (beginning with “Nipah virus (NiV) is an emergent zoonotic pathogen…”; includes human symptoms, sequelae, relapse, knowledge gaps) |
| Objectives | 3 | State specific objectives | Final paragraph of Introduction (“In this context, the present study aims to…”) |
| **Methods** | | | |
| Study design | 4 | Present key elements of study design early | Methods → *Study sites and population*, Paragraph 1 (“This cross-sectional study was conducted…”) |
| Setting | 5 | Describe setting and dates | Methods → *Study sites and population*, Paragraph 1 (“…conducted from November 2021 to February 2022, covering 23 districts…”) |
| Participants | 6(a) | Eligibility criteria, sources, and methods of participant selection | Methods → *Nipah virus infection survivors in Bangladesh* (definition of total survivors) + *Definition of survivor* + *Study sites and population*, Paragraph 2 (“From the identified 85 survivors…54 screened…31 excluded…52 enrolled…”) |
| Variables | 7 | Clearly define outcomes, exposures, predictors | Methods → *Definition of survivor*, Table 1; *Clinical and functional assessment* (“…documented new-onset symptoms…” + WG-ES domains) |
| Data sources/  measurement | 8 | Data sources and measurement; comparability | Methods → *Clinical evaluation* Paragraphs 1–2 (“A questionnaire was administered by trained physicians…”; “data were cross-checked…”; “WG-ES administered…”) |
| Bias | 9 | Describe efforts to address bias | Discussion → Limitations Paragraph (“…recall bias… mitigated by cross-checking with surveillance records…”) |
| Study size | 10 | Explain study size | Methods → *Study sites and population*, Paragraph 2 (“52 survivors enrolled…based on entire eligible survivor population over 18 years…”) |
| Quantitative variables | 11 | Handling of quantitative variables | Methods → *Statistical analysis* (Paragraph beginning “Data were analyzed using…”—describes descriptive summary methods used) |
| Statistical methods | 12 (a-e) | Statistical methods; confounding; missing data; subgroup methods | Methods → Statistical analysis section—describes descriptive analyses; no confounder adjustment applicable for design; missing data negligible and noted in Results Table footnotes |
| **Results** | | | |
| Participants | 13(a) | Report numbers at each stage | Results → First paragraph under *Survivor characteristics* (“…85 survivors identified…54 screened…52 enrolled…”) |
|  | 13(b) | Reasons for non-participation | Methods → *Study sites and population*, Paragraph 2 (“…31 excluded due to age…2 excluded for acute febrile illness…”) |
|  | 13(c) | Flow diagram | Appendix (Study population distribution) |
| Descriptive data | 14(a) | Descriptive characteristics | Results → Table 1 & Table 2; narrative in Paragraph 1 under *Survivor characteristics* |
|  | 14(b) | Indicate missing data | Tables 3–4 include “n = …” with denominators; no significant missing data |
| Outcome data | 15 | Outcome data | Results → Sections beginning “Post-infection, survivors reported…”; Tables 3 & 4 |
| Main results | 16(a–c) | Main results; estimates; precision | Results → Entire Results; descriptive statistics reported; no adjusted estimates applicable |
| Other analyses | 17 | Subgroup / sensitivity analyses | Not applicable (full survivor population assessed; no subgroups defined) |
| **Discussion** | | | |
| Key results | 18 | Summarize key results | Discussion → Opening paragraph (“This study provides evidence of the sequelae…”) |
| Limitations | 19 | Discuss limitations | Discussion → *Limitations Paragraph* (“Our study faced several limitations…”; includes recall bias, imaging limitations, lack of comparator group) |
| Interpretation | 20 | Interpretation considering limitations & context | Discussion → Multiple paragraphs comparing findings with Malaysia, Singapore, Bangladesh, India; paragraph beginning “Given that the Nipah virus has historically caused…” |
| Generalisability | 21 | Generalizability | Discussion → Paragraph discussing strain variation, survivor heterogeneity, healthcare differences (“In light of these findings…further investigation warranted…”) |
| **Other information** | | | |
| Funding | 22 | Funding and role of funders | Funding Statement at end of manuscript (“This work was supported by…”; funder involvement clarified) |

Supplementary table 2: Demographic and clinical findings during active Nipah virus infection

| **Characteristics** | **n** | **% [95% CI]** |
| --- | --- | --- |
| Male Sex | 28/52 | 54 [40, 67] |
| Fever | 52/52 | 100 [93, 100] |
| Headache | 37/52 | 71 [57, 83] |
| Severe Weakness | 35/52 | 67 [53, 80] |
| Nausea and vomiting | 28/52 | 54 [39, 68] |
| Drowsiness | 20/45 | 44 [30, 60] |
| Altered mental status | 20/45 | 44 [30, 60] |
| Cough | 20/52 | 38 [25, 53] |
| Convulsion | 17/52 | 33 [20, 47] |
| Muscle aches | 16/48 | 33 [20, 48] |
| Breathing difficulty | 17/52 | 31 [19, 45] |
| Joint pain | 10/45 | 22 [11, 37] |
| Irritability | 10/45 | 22 [11, 37] |
| Sore throat | 9/45 | 20 [10, 35] |
| Diarrhea | 10/52 | 19 [10, 33] |
| Neck stiffness | 8/45 | 18 [8, 32] |
| Rash | 5/48 | 10 [3, 23] |

Supplementary table 3: Association of the severity and chronicity of post infection signs/symptoms with sex, age in years at the time of acute infection and age in years during study period

| **Symptom** | **Category** | **Level** | **Sex** | | | **Age in years at acute infection** | | | **Age in years during study period** | | | |
| --- | --- | --- | --- | --- | --- | --- | --- | --- | --- | --- | --- | --- |
|  |  |  | **Female (N=24)** | **Male (N=28)** | **p-value** | **Age<=21** | **Age>=22** | **p-value** | **<=25 (N=12)** | **26–40 (N=24)** | **>40 (N=16)** | **p-value** |
| **Fever** | Severity | Mild | 8 (38) | 12 (55) | 0.48 | 10 (53) | 10 (42) | 0.16 | 6 (60) | 9 (47) | 5 (36) | 0.72 |
|  |  | Moderate | 5 (24) | 5 (23) |  | 6 (32) | 4 (17) |  | 2 (20) | 5 (26) | 3 (21) |  |
|  |  | Severe | 8 (38) | 5 (23) |  | 3 (16) | 10 (42) |  | 2 (20) | 5 (26) | 6 (43) |  |
|  | Chronicity | Acute | 2 (10) | 5 (23) | 0.44 | 2 (11) | 5 (21) | 0.1 | 1 (10) | 5 (26) | 1 (7) | 0.34 |
|  |  | Chronic | 2 (10) | 1 (5) |  | 3 (16) | 0 (0) |  | 1 (10) | 2 (11) | 0 (0) |  |
|  |  | Episodic | 17 (81) | 16 (73) |  | 14 (74) | 19 (79) |  | 8 (80) | 12 (63) | 13 (93) |  |
| **Headaches** | Severity | Mild | 3 (17) | 14 (64) | 0.009 | 9 (47) | 8 (38) | 0.83 | 3 (27) | 10 (59) | 4 (33) | 0.41 |
|  |  | Moderate | 7 (39) | 5 (23) |  | 5 (26) | 7 (33) |  | 5 (45) | 3 (18) | 4 (33) |  |
|  |  | Severe | 8 (44) | 3 (14) |  | 5 (26) | 6 (29) |  | 3 (27) | 4 (24) | 4 (33) |  |
|  | Chronicity | Acute | 0 (0) | 1 (5) | 0.15 | 0 (0) | 1 (5) | 0.28 | 0 (0) | 1 (6) | 0 (0) | 0.77 |
|  |  | Chronic | 8 (44) | 4 (18) |  | 4 (21) | 8 (38) |  | 4 (36) | 4 (24) | 4 (33) |  |
|  |  | Episodic | 10 (56) | 17 (77) |  | 15 (79) | 12 (57) |  | 7 (64) | 12 (71) | 8 (67) |  |
| **Altered mental status** | Severity | Mild | 8 (67) | 4 (40) | 0.34 | 5 (56) | 7 (54) | 0.18 | 2 (40) | 8 (80) | 2 (29) | 0.11 |
|  |  | Moderate | 3 (25) | 3 (30) |  | 1 (11) | 5 (38) |  | 1 (20) | 1 (10) | 4 (57) |  |
|  |  | Severe | 1 (8) | 3 (30) |  | 3 (33) | 1 (8) |  | 2 (40) | 1 (10) | 1 (14) |  |
|  | Chronicity | Chronic | 3 (25) | 7 (70) | 0.035 | 7 (78) | 3 (23) | 0.011 | 5 (100) | 3 (30) | 2 (29) | 0.021 |
|  |  | Episodic | 9 (75) | 3 (30) |  | 2 (22) | 10 (77) |  | 0 (0) | 7 (70) | 5 (71) |  |
| **Loss of consciousness** | Severity | Mild | 2 (33) | 2 (33) | 1 | 2 (40) | 2 (29) | 0.11 | 1 (100) | 2 (29) | 1 (25) | 0.41 |
|  |  | Moderate | 1 (17) | 1 (17) |  | 2 (40) | 0 (0) |  | 0 (0) | 2 (29) | 0 (0) |  |
|  |  | Severe | 3 (50) | 3 (50) |  | 1 (20) | 5 (71) |  | 0 (0) | 3 (43) | 3 (75) |  |
|  | Chronicity | Acute | 1 (17) | 1 (17) | 0.79 | 2 (40) | 0 (0) | 0.084 | 0 (0) | 2 (29) | 0 (0) | 0.5 |
|  |  | Chronic | 2 (33) | 1 (17) |  | 0 (0) | 3 (43) |  | 0 (0) | 1 (14) | 2 (50) |  |
|  |  | Episodic | 3 (50) | 4 (67) |  | 3 (60) | 4 (57) |  | 1 (100) | 4 (57) | 2 (50) |  |
| **Seizures** | Severity | Mild | 0 (0) | 2 (40) | 0.22 | 1 (25) | 1 (17) | 0.73 | 0 (0) | 1 (25) | 1 (20) | 0.76 |
|  |  | Moderate | 3 (60) | 1 (20) |  | 2 (50) | 2 (33) |  | 0 (0) | 2 (50) | 2 (40) |  |
|  |  | Severe | 2 (40) | 2 (40) |  | 1 (25) | 3 (50) |  | 1 (100) | 1 (25) | 2 (40) |  |
|  | Chronicity | Acute | 1 (20) | 0 (0) | 0.29 | 1 (25) | 0 (0) | 0.2 | 0 (0) | 1 (25) | 0 (0) | 0.43 |
|  |  | Episodic | 4 (80) | 5 (100) |  | 3 (75) | 6 (100) |  | 1 (100) | 3 (75) | 5 (100) |  |
| **Sensory loss/changes** | Severity | Mild | 3 (75) | 1 (33) | 0.39 | 0 (0) | 4 (80) | 0.1 | 0 (0) | 3 (75) | 1 (100) | 0.31 |
|  |  | Moderate | 0 (0) | 1 (33) |  | 1 (50) | 0 (0) |  | 1 (50) | 0 (0) | 0 (0) |  |
|  |  | Severe | 1 (25) | 1 (33) |  | 1 (50) | 1 (20) |  | 1 (50) | 1 (25) | 0 (0) |  |
|  | Chronicity | Chronic | 3 (75) | 2 (67) | 0.81 | 1 (50) | 4 (80) | 0.43 | 1 (50) | 3 (75) | 1 (100) | 0.65 |
|  |  | Episodic | 1 (25) | 1 (33) |  | 1 (50) | 1 (20) |  | 1 (50) | 1 (25) | 0 (0) |  |
| **Depressed mood** | Severity | Mild | 7 (58) | 5 (71) | 0.52 | 4 (57) | 8 (67) | 0.3 | 2 (50) | 6 (75) | 4 (57) | 0.29 |
|  |  | Moderate | 2 (17) | 0 (0) |  | 0 (0) | 2 (17) |  | 0 (0) | 0 (0) | 2 (29) |  |
|  |  | Severe | 3 (25) | 2 (29) |  | 3 (43) | 2 (17) |  | 2 (50) | 2 (25) | 1 (14) |  |
|  | Chronicity | Chronic | 3 (25) | 3 (43) | 0.42 | 3 (43) | 3 (25) | 0.42 | 2 (50) | 2 (25) | 2 (29) | 0.66 |
|  |  | Episodic | 9 (75) | 4 (57) |  | 4 (57) | 9 (75) |  | 2 (50) | 6 (75) | 5 (71) |  |
| **Pain behind the eyes** | Severity | Mild | 3 (30) | 4 (80) | 0.16 | 3 (75) | 4 (36) | 0.34 | 2 (100) | 4 (80) | 1 (13) | 0.071 |
|  |  | Moderate | 4 (40) | 1 (20) |  | 1 (25) | 4 (36) |  | 0 (0) | 1 (20) | 4 (50) |  |
|  |  | Severe | 3 (30) | 0 (0) |  | 0 (0) | 3 (27) |  | 0 (0) | 0 (0) | 3 (38) |  |
|  | Chronicity | Chronic | 2 (20) | 1 (20) | 1 | 0 (0) | 3 (27) | 0.24 | 0 (0) | 1 (20) | 2 (25) | 0.73 |
|  |  | Episodic | 8 (80) | 4 (80) |  | 4 (100) | 8 (73) |  | 2 (100) | 4 (80) | 6 (75) |  |
| **Blurry vision** | Severity | Mild | 5 (38) | 4 (33) | 0.97 | 5 (71) | 4 (22) | 0.068 | 2 (67) | 6 (50) | 1 (10) | 0.19 |
|  |  | Moderate | 5 (38) | 5 (42) |  | 1 (14) | 9 (50) |  | 0 (0) | 4 (33) | 6 (60) |  |
|  |  | Severe | 3 (23) | 3 (25) |  | 1 (14) | 5 (28) |  | 1 (33) | 2 (17) | 3 (30) |  |
|  | Chronicity | Chronic | 10 (77) | 9 (75) | 0.91 | 5 (71) | 14 (78) | 0.74 | 2 (67) | 9 (75) | 8 (80) | 0.89 |
|  |  | Episodic | 3 (23) | 3 (25) |  | 2 (29) | 4 (22) |  | 1 (33) | 3 (25) | 2 (20) |  |
| **Other visual disturbances** | Severity | Mild | 3 (75) | 7 (88) | 0.58 | 6 (75) | 4 (100) | 0.27 | 3 (60) | 6 (100) | 1 (100) | 0.19 |
|  |  | Moderate | 1 (25) | 1 (13) |  | 2 (25) | 0 (0) |  | 2 (40) | 0 (0) | 0 (0) |  |
|  | Chronicity | Acute | 0 (0) | 1 (13) | 0.75 | 1 (13) | 0 (0) | 0.22 | 1 (20) | 0 (0) | 0 (0) | 0.52 |
|  |  | Chronic | 1 (25) | 2 (25) |  | 3 (38) | 0 (0) |  | 2 (40) | 1 (17) | 0 (0) |  |
|  |  | Episodic | 3 (75) | 5 (63) |  | 4 (50) | 4 (100) |  | 2 (40) | 5 (83) | 1 (100) |  |
| **Hearing loss** | Severity | Mild | 2 (67) | 1 (50) | 0.71 | 1 (100) | 2 (50) | 0.36 | – | 1 (100) | 2 (50) | 0.36 |
|  |  | Moderate | 1 (33) | 1 (50) |  | 0 (0) | 2 (50) |  | – | 0 (0) | 2 (50) |  |
|  | Chronicity | Chronic | 1 (33) | 2 (100) | 0.14 | 1 (100) | 2 (50) | 0.36 | – | 1 (100) | 2 (50) | 0.36 |
|  |  | Episodic | 2 (67) | 0 (0) |  | 0 (0) | 2 (50) |  | – | 0 (0) | 2 (50) |  |
| **Fatigue** | Severity | Mild | 4 (24) | 5 (50) | 0.084 | 6 (67) | 3 (17) | 0.034 | 3 (50) | 4 (50) | 2 (15) | 0.37 |
|  |  | Moderate | 7 (41) | 5 (50) |  | 2 (22) | 10 (56) |  | 2 (33) | 2 (25) | 8 (62) |  |
|  |  | Severe | 6 (35) | 0 (0) |  | 1 (11) | 5 (28) |  | 1 (17) | 2 (25) | 3 (23) |  |
|  | Chronicity | Chronic | 9 (53) | 7 (70) | 0.38 | 6 (67) | 10 (56) | 0.58 | 3 (50) | 5 (63) | 8 (62) | 0.87 |
|  |  | Episodic | 8 (47) | 3 (30) |  | 3 (33) | 8 (44) |  | 3 (50) | 3 (38) | 5 (38) |  |
| **Trouble sleeping** | Severity | Mild | 1 (8) | 1 (14) | 0.91 | 1 (14) | 1 (8) | 0.6 | 0 (0) | 2 (20) | 0 (0) | 0.73 |
|  |  | Moderate | 7 (58) | 4 (57) |  | 3 (43) | 8 (67) |  | 2 (67) | 5 (50) | 4 (67) |  |
|  |  | Severe | 4 (33) | 2 (29) |  | 3 (43) | 3 (25) |  | 1 (33) | 3 (30) | 2 (33) |  |
|  | Chronicity | Acute | 1 (8) | 0 (0) | 0.58 | 0 (0) | 1 (8) | 0.58 | 0 (0) | 1 (10) | 0 (0) | 0.92 |
|  |  | Chronic | 8 (67) | 4 (57) |  | 4 (57) | 8 (67) |  | 2 (67) | 6 (60) | 4 (67) |  |
|  |  | Episodic | 3 (25) | 3 (43) |  | 3 (43) | 3 (25) |  | 1 (33) | 3 (30) | 2 (33) |  |
| **Cough** | Severity | Mild | 10 (63) | 11 (65) | 0.84 | 10 (67) | 11 (61) | 0.53 | 6 (75) | 7 (54) | 8 (67) | 0.85 |
|  |  | Moderate | 4 (25) | 3 (18) |  | 2 (13) | 5 (28) |  | 1 (13) | 4 (31) | 2 (17) |  |
|  |  | Severe | 2 (13) | 3 (18) |  | 3 (20) | 2 (11) |  | 1 (13) | 2 (15) | 2 (17) |  |
|  | Chronicity | Acute | 1 (6) | 1 (6) | 0.32 | 1 (7) | 1 (6) | 0.41 | 0 (0) | 2 (15) | 0 (0) | 0.4 |
|  |  | Chronic | 2 (13) | 0 (0) |  | 0 (0) | 2 (11) |  | 0 (0) | 1 (8) | 1 (8) |  |
|  |  | Episodic | 13 (81) | 16 (94) |  | 14 (93) | 15 (83) |  | 8 (100) | 10 (77) | 11 (92) |  |
| **Shortness of breath** | Severity | Mild | 2 (25) | 1 (33) | 0.63 | 1 (50) | 2 (22) | 0.63 | 1 (50) | 0 (0) | 2 (29) | 0.56 |
|  |  | Moderate | 4 (50) | 2 (67) |  | 1 (50) | 5 (56) |  | 1 (50) | 2 (100) | 3 (43) |  |
|  |  | Severe | 2 (25) | 0 (0) |  | 0 (0) | 2 (22) |  | 0 (0) | 0 (0) | 2 (29) |  |
|  | Chronicity | Chronic | 1 (13) | 0 (0) | 0.52 | 0 (0) | 1 (11) | 0.62 | 0 (0) | 0 (0) | 1 (14) | 0.73 |
|  |  | Episodic | 7 (88) | 3 (100) |  | 2 (100) | 8 (89) |  | 2 (100) | 2 (100) | 6 (86) |  |
| **Arm weakness** | Severity | Mild | 7 (50) | 6 (67) | 0.42 | 5 (63) | 8 (53) | 0.51 | 3 (60) | 5 (56) | 5 (56) | 0.81 |
|  |  | Moderate | 2 (14) | 2 (22) |  | 2 (25) | 2 (13) |  | 0 (0) | 2 (22) | 2 (22) |  |
|  |  | Severe | 5 (36) | 1 (11) |  | 1 (13) | 5 (33) |  | 2 (40) | 2 (22) | 2 (22) |  |
|  | Chronicity | Acute | 1 (7) | 0 (0) | 0.65 | 0 (0) | 1 (7) | 0.76 | 0 (0) | 1 (11) | 0 (0) | 0.74 |
|  |  | Chronic | 7 (50) | 4 (44) |  | 4 (50) | 7 (47) |  | 3 (60) | 4 (44) | 4 (44) |  |
|  |  | Episodic | 6 (43) | 5 (56) |  | 4 (50) | 7 (47) |  | 2 (40) | 4 (44) | 5 (56) |  |
| **Leg weakness** | Severity | Mild | 3 (23) | 2 (33) | 0.46 | 1 (25) | 4 (27) | 0.8 | 1 (25) | 0 (0) | 4 (50) | 0.047 |
|  |  | Moderate | 6 (46) | 1 (17) |  | 2 (50) | 5 (33) |  | 3 (75) | 2 (29) | 2 (25) |  |
|  |  | Severe | 4 (31) | 3 (50) |  | 1 (25) | 6 (40) |  | 0 (0) | 5 (71) | 2 (25) |  |
|  | Chronicity | Acute | 1 (8) | 0 (0) | 0.44 | 0 (0) | 1 (7) | 0.8 | 0 (0) | 1 (14) | 0 (0) | 0.45 |
|  |  | Chronic | 7 (54) | 5 (83) |  | 3 (75) | 9 (60) |  | 3 (75) | 5 (71) | 4 (50) |  |
|  |  | Episodic | 5 (38) | 1 (17) |  | 1 (25) | 5 (33) |  | 1 (25) | 1 (14) | 4 (50) |  |
| **Muscle pain** | Severity | Mild | 6 (50) | 6 (67) | 0.67 | 7 (78) | 5 (42) | 0.25 | 5 (83) | 4 (57) | 3 (38) | 0.42 |
|  |  | Moderate | 3 (25) | 1 (11) |  | 1 (11) | 3 (25) |  | 0 (0) | 2 (29) | 2 (25) |  |
|  |  | Severe | 3 (25) | 2 (22) |  | 1 (11) | 4 (33) |  | 1 (17) | 1 (14) | 3 (38) |  |
|  | Chronicity | Chronic | 2 (17) | 1 (11) | 0.72 | 0 (0) | 3 (25) | 0.11 | 1 (17) | 1 (14) | 1 (13) | 0.98 |
|  |  | Episodic | 10 (83) | 8 (89) |  | 9 (100) | 9 (75) |  | 5 (83) | 6 (86) | 7 (88) |  |
| **Joint pain** | Severity | Mild | 6 (50) | 8 (67) | 0.53 | 8 (80) | 6 (43) | 0.18 | 4 (80) | 6 (67) | 4 (40) | 0.43 |
|  |  | Moderate | 3 (25) | 1 (8) |  | 1 (10) | 3 (21) |  | 0 (0) | 2 (22) | 2 (20) |  |
|  |  | Severe | 3 (25) | 3 (25) |  | 1 (10) | 5 (36) |  | 1 (20) | 1 (11) | 4 (40) |  |
|  | Chronicity | Acute | 0 (0) | 1 (8) | 0.5 | 1 (10) | 0 (0) | 0.2 | 0 (0) | 1 (11) | 0 (0) | 0.55 |
|  |  | Chronic | 4 (33) | 5 (42) |  | 2 (20) | 7 (50) |  | 1 (20) | 3 (33) | 5 (50) |  |
|  |  | Episodic | 8 (67) | 6 (50) |  | 7 (70) | 7 (50) |  | 4 (80) | 5 (56) | 5 (50) |  |
| **Diarrhea** | Severity | Mild | 7 (88) | 11 (79) | 0.73 | 10 (91) | 8 (73) | 0.46 | 5 (83) | 6 (86) | 7 (78) | 0.45 |
|  |  | Moderate | 1 (13) | 2 (14) |  | 1 (9) | 2 (18) |  | 1 (17) | 0 (0) | 2 (22) |  |
|  |  | Severe | 0 (0) | 1 (7) |  | 0 (0) | 1 (9) |  | 0 (0) | 1 (14) | 0 (0) |  |
|  | Chronicity | Episodic | 8 (100) | 14 (100) | - | 11 (100) | 11 (100) | - | 6 (100) | 7 (100) | 9 (100) | - |
| **Abdominal pain** | Severity | Mild | 6 (60) | 7 (58) | 0.89 | 6 (75) | 7 (50) | 0.46 | 3 (75) | 5 (71) | 5 (45) | 0.7 |
|  |  | Moderate | 1 (10) | 2 (17) |  | 1 (13) | 2 (14) |  | 0 (0) | 1 (14) | 2 (18) |  |
|  |  | Severe | 3 (30) | 3 (25) |  | 1 (13) | 5 (36) |  | 1 (25) | 1 (14) | 4 (36) |  |
|  | Chronicity | Chronic | 1 (10) | 1 (8) | 0.89 | 1 (13) | 1 (7) | 0.67 | 0 (0) | 1 (14) | 1 (9) | 0.73 |
|  |  | Episodic | 9 (90) | 11 (92) |  | 7 (88) | 13 (93) |  | 4 (100) | 6 (86) | 10 (91) |  |
| **Weight loss** | Severity | Mild | 5 (63) | 4 (80) | 0.47 | 3 (60) | 6 (75) | 0.1 | 2 (100) | 3 (60) | 4 (67) | 0.21 |
|  |  | Moderate | 2 (25) | 0 (0) |  | 0 (0) | 2 (25) |  | 0 (0) | 0 (0) | 2 (33) |  |
|  |  | Severe | 1 (13) | 1 (20) |  | 2 (40) | 0 (0) |  | 0 (0) | 2 (40) | 0 (0) |  |
|  | Chronicity | Acute | 2 (25) | 0 (0) | 0.1 | 1 (20) | 1 (13) | 0.12 | 0 (0) | 2 (40) | 0 (0) | 0.055 |
|  |  | Chronic | 0 (0) | 2 (40) |  | 2 (40) | 0 (0) |  | 0 (0) | 2 (40) | 0 (0) |  |
|  |  | Episodic | 6 (75) | 3 (60) |  | 2 (40) | 7 (88) |  | 2 (100) | 1 (20) | 6 (100) |  |
| **Persistent ataxia/gait problems** | Severity | Mild | 1 (13) | 2 (40) | 0.15 | 1 (33) | 2 (20) | 0.72 | 1 (50) | 1 (17) | 1 (20) | 0.25 |
|  |  | Moderate | 6 (75) | 1 (20) |  | 1 (33) | 6 (60) |  | 1 (50) | 2 (33) | 4 (80) |  |
|  |  | Severe | 1 (13) | 2 (40) |  | 1 (33) | 2 (20) |  | 0 (0) | 3 (50) | 0 (0) |  |
|  | Chronicity | Chronic | 5 (63) | 5 (100) | 0.12 | 3 (100) | 7 (70) | 0.28 | 2 (100) | 5 (83) | 3 (60) | 0.46 |
|  |  | Episodic | 3 (38) | 0 (0) |  | 0 (0) | 3 (30) |  | 0 (0) | 1 (17) | 2 (40) |  |
| **Personality/behavior changes** | Severity | Mild | 5 (36) | 1 (13) | 0.33 | 1 (13) | 5 (36) | 0.46 | 2 (40) | 1 (14) | 3 (30) | 0.49 |
|  |  | Moderate | 8 (57) | 5 (63) |  | 6 (75) | 7 (50) |  | 2 (40) | 6 (86) | 5 (50) |  |
|  |  | Severe | 1 (7) | 2 (25) |  | 1 (13) | 2 (14) |  | 1 (20) | 0 (0) | 2 (20) |  |
|  | Chronicity | Chronic | 4 (29) | 7 (88) | 0.008 | 6 (75) | 5 (36) | 0.076 | 3 (60) | 4 (57) | 4 (40) | 0.69 |
|  |  | Episodic | 10 (71) | 1 (13) |  | 2 (25) | 9 (64) |  | 2 (40) | 3 (43) | 6 (60) |  |
| **Memory/concentration problems** | Severity | Mild | 4 (24) | 6 (55) | 0.097 | 6 (60) | 4 (22) | 0.11 | 3 (50) | 5 (45) | 2 (18) | 0.15 |
|  |  | Moderate | 10 (59) | 2 (18) |  | 2 (20) | 10 (56) |  | 3 (50) | 2 (18) | 7 (64) |  |
|  |  | Severe | 3 (18) | 3 (27) |  | 2 (20) | 4 (22) |  | 0 (0) | 4 (36) | 2 (18) |  |
|  | Chronicity | Acute | 1 (6) | 0 (0) | 0.61 | 0 (0) | 1 (6) | 0.72 | 0 (0) | 1 (9) | 0 (0) | 0.53 |
|  |  | Chronic | 10 (59) | 8 (73) |  | 7 (70) | 11 (61) |  | 5 (83) | 7 (64) | 6 (55) |  |
|  |  | Episodic | 6 (35) | 3 (27) |  | 3 (30) | 6 (33) |  | 1 (17) | 3 (27) | 5 (45) |  |
| **Myoclonus** | Severity | Mild | 6 (60) | 8 (53) | 0.3 | 7 (58) | 7 (54) | 0.083 | 3 (43) | 9 (75) | 2 (33) | 0.13 |
|  |  | Moderate | 0 (0) | 3 (20) |  | 3 (25) | 0 (0) |  | 1 (14) | 2 (17) | 0 (0) |  |
|  |  | Severe | 4 (40) | 4 (27) |  | 2 (17) | 6 (46) |  | 3 (43) | 1 (8) | 4 (67) |  |
|  | Chronicity | Acute | 1 (10) | 0 (0) | 0.11 | 0 (0) | 1 (8) | 0.34 | 0 (0) | 1 (8) | 0 (0) | 0.58 |
