# Supplementary material for: Long-term sequelae and functional outcomes in the largest cohort of Nipah virus survivors in Bangladesh
Source: Lancet Reg Health Southeast Asia. 2026 Feb 11;45:100729. doi: 10.1016/j.lansea.2026.100729 (PMC12915286; doi:10.1016/j.lansea.2026.100729)
Supplement: Appendix [file mmc2.docx]

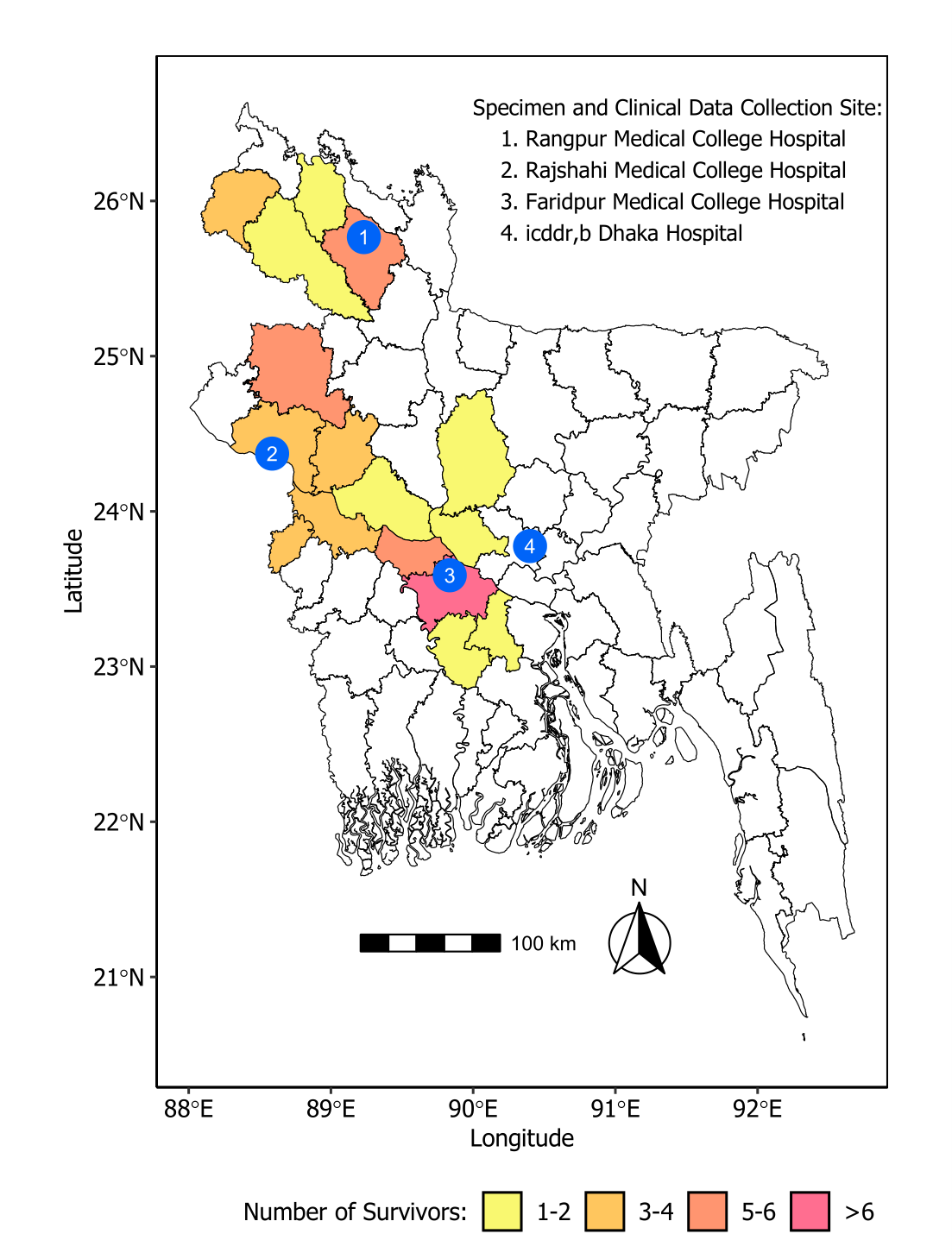


Appendix: Locations of Nipah infection survivors and adjacent sentinel hospitals of national Nipah surveillance/healthcare facilities, where clinical evaluation was performed
